# Carborane-Containing Iron Oxide@Gold Nanoparticles for Potential Application in Neutron Capture Therapy

**DOI:** 10.3390/nano15161243

**Published:** 2025-08-13

**Authors:** Zhangali A. Bekbol, Kairat A. Izbasar, Alexander Zaboronok, Lana I. Lissovskaya, Haolan Yang, Yuriy Pihosh, Eiichi Ishikawa, Rafael I. Shakirzyanov, Ilya V. Korolkov

**Affiliations:** 1 The Institute of Nuclear Physics, Ibragimov Str. 1, 050032 Almaty, Kazakhstan; zhangali.bekbol@mail.ru (Z.A.B.); ms.defrance@mail.ru (L.I.L.); i.korolkov@inp.kz (I.V.K.); 2 Engineering Profile Laboratory, L.N. Gumilyov Eurasian National University, Satpaev Str. 5, 010008 Astana, Kazakhstan; shakirzyanov_ri@enu.kz; 3 Department of Neurosurgery, Institute of Medicine, University of Tsukuba, 1-1-1 Tennodai, Tsukuba 305-8575, Ibaraki, Japan; s2430438@u.tsukuba.ac.jp (H.Y.); e-ishikawa@md.tsukuba.ac.jp (E.I.); 4 Office of University Professors, The University of Tokyo, 2-11-16 Yayoi, Bunkyo-ku, Tokyo 113-8656, Japan; pihosh_y@arpchem.t.u-tokyo.ac.jp

**Keywords:** nanoparticles, iron oxide, drug delivery, carborane, neutron capture therapy

## Abstract

Cancer remains one of the most pressing global health challenges, driving the need for innovative treatment strategies. Boron neutron capture therapy (BNCT) offers a highly selective approach to destroying cancer cells while sparing healthy tissues. To improve boron delivery, Fe_3_O_4_@Au nanoparticles were developed and functionalized with a boron-containing carborane compound. Fe_3_O_4_ nanoparticles were synthesized and covered by gold, followed by (3-Aminopropyl)triethoxysilane (APTES) modification to introduce amino groups for carborane immobilization. Comprehensive characterization using SEM, DLS, FTIR, EDX, Brunauer–Emmett–Teller (BET), and XRD confirmed successful functionalization at each stage. TEM confirmed the final structure and elemental composition of the nanoparticles. BET analysis revealed a surface area of 94.69 m^2^/g and a pore volume of 0.51 cm^3^/g after carborane loading. Initial release studies in PBS demonstrated the removal of only loosely bound carborane within 48 h, with FTIR confirming stable retention of the compound on the nanoparticle surface. The modified nanoparticles achieved a stable zeta potential of −20 mV. The particles showed low toxicity within a range of concentrations (0–300 μg Fe/mL) and were efficiently accumulated by U251MG cells. These results demonstrate the potential of the obtained nanoparticles to carry boron and gold for their possible application as a theranostic agent.

## 1. Introduction

Cancer remains a significant universal health challenge, ranking as the second leading cause of death and contributing substantially to the worldwide disease burden [1]. In 2022, approximately 20 million new cancer cases were reported globally, with more than 9.7 million deaths attributed to the disease [2].

Traditional cancer therapies, including surgery, chemotherapy, and radiation therapy, have proven effective in many cases. However, these methods often struggle to treat gliomas, sarcomas, and adenocarcinomas [3]. Gliomas, for instance, exhibit high intrinsic resistance to radiation, limiting therapeutic outcomes [4]. Furthermore, these treatments frequently damage healthy tissues, leading to severe side effects and restricting their applicability.

In boron neutron capture therapy (BNCT), a compound containing boron-10 (^10^B) is typically delivered to the patient through intravenous infusion. Without neutron irradiation, the boron compound remains non-toxic and non-radioactive, allowing it to selectively accumulate within cancer cells. Over time, the boron becomes concentrated in the tumor, while healthy tissues retain significantly lower boron levels. The tumor area is then exposed to an epithermal neutron beam, which slows down within the tissue and interacts with the ^10^B nuclei. This reaction produces high linear energy transfer (LET) alpha particles and lithium nuclei, which destroy cancer cells in the immediate vicinity while sparing surrounding healthy tissues [5,6,7,8,9]. BNCT has recently gained clinical approval in Japan, utilizing a hospital-based accelerator neutron source (Sumitomo Heavy Industries) and L-boronophenylalanine (Steboronine^®^, Stella Pharma, Osaka, Japan) [10,11]. This treatment is now reimbursed under the national health insurance system for select malignancies. Another accelerator-based neutron source is being tested for clinical application at the University of Tsukuba [12,13]. The development of accelerators for BNCT promoted the research and development of this technology worldwide. However, a significant challenge still lies in efficiently delivering boron compounds to tumors. Ensuring selective accumulation in cancer cells while avoiding healthy tissues is crucial for the success of BNCT.

Various methods have been developed to deliver boron-rich compounds directly to cancerous tumors, including liposomes [14,15], gold nanoparticles [16,17], boron nitride nanotubes [18,19], chitosan [20], and magnetic nanostructures [21,22,23]. The key requirements for delivery agents include precision targeting, low toxicity, stability, effective accumulation in the body, rapid clearance, and the ability to achieve a sufficient boron concentration at the tumor site. Among these options, magnetic nanoparticles stand out as a promising approach due to their magnetic properties, which enable precise localization in specific areas. Magnetic nanoparticles have properties suitable for such medical applications [24,25,26,27], for example, as their superparamagnetic nature allows for use as contrast agents in MRI for better tissue visualization [28]. The biomedical applicability of these materials largely depends on their stability, biocompatibility, and precise control over their size, morphology, and surface characteristics during synthesis. In a previous study, our team synthesized a Gd-DTPA carborane-containing compound and successfully immobilized it on the surface of modified magnetic nanoparticles [23]. However, it was necessary to increase the concentration of the boron-containing compound, and a simplified method was implemented to achieve this enhancement.

In this study, nanoparticles were modified with a gold coating to form a core–shell structure on their surface, as previously described [29]. Subsequently, Fe_3_O_4_@Au nanoparticles were functionalized with (3-aminopropyl)triethoxysilane (APTES); essentially, a boron-containing carborane compound was immobilized through the protonation of silane groups using hydrochloric acid in solution.

The addition of a gold shell offers several advantages, including enhanced energy deposition. Incorporating heavy elements, such as gold, into tumors significantly increases the cross-section for the photoelectric effect compared to lighter nuclei. This leads to a rise in localized energy absorption, creating microscopic regions with intensified dose. Additionally, gold nuclei (^197^Au) can undergo activation during neutron irradiation, forming ^198^Au [30], which emits β-particles. These particles contribute to the destruction of tumor cells, further enhancing therapeutic efficacy. Additionally, the γ-rays emitted by ^198^Au can be detected externally using a gamma spectrometer, enabling verification of the absorbed dose [31].

## 2. Materials and Methods

### 2.1. Materials

Iron(II) chloride tetrahydrate and iron(III) chloride hexahydrate were purchased from Sigma-Aldrich (Merck KGaA, Darmstadt, Germany). (3-Aminopropyl)triethoxysilane (APTES) and gold(III) chloride trihydrate were purchased from Sigma-Aldrich (St. Louis, MO, USA). Sodium hydroxide, hydrochloric acid (HCl), citric acid, and ethanol were of analytical grade. Deionized water (18.2 MΩ·cm) was used in all experiments.

### 2.2. Synthesis of Fe_3_O_4_ Nanoparticles

A solution was prepared by mixing 100 mL of deionized water with 11.8 mL of HCl, followed by the addition of 9.94 g of FeCl_2_·4H_2_O and 27.03 g of FeCl_3_·6H_2_O. The reaction was conducted under an argon atmosphere at 80 °C. After 2 h of vertical stirring, the resulting black nanoparticles were magnetically collected and subsequently washed with ethanol and deionized water. The nanoparticles were then dried in a Petri dish at 60 °C under ambient air conditions.

### 2.3. Modification of Fe_3_O_4_ NPs by Covering with Gold

Gold coating of Fe_3_O_4_ NPs was performed following a method described elsewhere [29]. Briefly, 1 g of Fe_3_O_4_ nanoparticles was first functionalized with citric acid by dispersing them in a 0.1 g/mL citric acid solution and stirring the mixture at 80 °C for 3 h. The nanoparticles were then magnetically separated and washed with deionized water. To facilitate gold complexation, the citric acid-coated nanoparticles were immersed in a 0.1% aqueous solution of gold(III) chloride for 1 h. Reduction of gold ions was achieved by adding sodium citrate, and the reaction was allowed to proceed at 60–70 °C for 3 h. The gold-coated nanoparticles were magnetically separated, washed with diluted hydrochloric acid to remove uncoated iron oxide particles, and subsequently purified.

### 2.4. Modification of Fe_3_O_4_@Au with APTES

An amount of 1 g of Fe_3_O_4_@Au nanoparticles was dispersed in 100 mL of deionized water, and (3-aminopropyl)triethoxysilane (APTES) was added to achieve a final concentration of 0.0126 M. The suspension was stirred at 80 °C for 5 h. Upon completion of the reaction, the nanoparticles were magnetically separated, extensively washed with deionized water, and dried.

### 2.5. Synthesis of Carborane-Containing Agent

Potassium 3-(2-isopropyl-1,2-dicarba-*closo*-dodecaboran-1-yl)-3-phenylpropanoate was synthesized according to the procedure described in [32]. First, 3-(2-isopropyl-1,2-dicarba-*closo*-dodecaboran-1-yl)-3-phenylpropanoic acid was obtained as follows: diethyl 2-[4-(1-isopropyl-1,2-dicarba-*closo*-dodecaboran-1-yl)benzyl]propanedioate (4.32 g, 0.01 mol), acetic acid (50 mL, 0.874 mol), and hydrobromic acid (20 mL, 0.368 mol) were added to a 250 mL round-bottom flask equipped with a magnetic stirrer and a reflux condenser. The reaction mixture was vigorously stirred and heated for 24 h, during which the solution gradually changed in color from light yellow to light brown. The progress of the reaction was monitored by thin-layer chromatography (TLC). Upon completion, a precipitate of off-white crystals formed and was collected by filtration through a Schott glass filter. The solid was thoroughly washed with deionized water, dried in a desiccator, and subsequently recrystallized to afford the pure product (yield: 1.89 g (60%); white crystals; mp 159–160.6 °C).

FTIR spectrum (ν, cm^−1^): 2700–3200 (O-H), 3030, 3063 (C-H, ar.), 2873–2981 (C-H), 2558, 2612 (B-H), 1715 (C=O, acid), 1457 (C=C, ring modes), 1214, 1267 (C-O), 1086, 1213 (in-plane, C-H), 698 (out-of-plane, C-H).

^1^H NMR (80 MHz, DMSO-*d*_6_): δ = 1.4–2.6 (m, 10H, B-H), 1.19 (d, *J* = 6.3 Hz, 6H, CH(CH_3_)_2_), 2.46 (q, *J* = 1.9 Hz, 1H, CH(CH_3_)_2_), 2.97 (d, *J* = 7.7 Hz, 2H, -CH_2_-), 3.86 (t, *J* = 7.7 Hz, 1H, C-H), 7.29 (s, 5H, C_sp2_H), 12.30 (s, 1H, -COOH).

^13^C NMR (20 MHz, DMSO-*d*_6_): δ = 24.58 (-CH_3_), 24.87 (-CH_3_), 30.78 (C-H), 41.50 (-CH_2_), 43.66 (C-H), 87.73 (C_4_, carborane core), 90.70 (C_4_, carborane core), 128.51 (C_sp2_H), 128.73 (C_sp2_H), 129.66 (C_sp2_H, 3 atoms), 139. 49 (C_4_, phenyl), 171.52 (C_4_, -COOH).

^11^B NMR (26 MHz, DMSO-*d*_6_): −4.6 (5B), −9.3 (5B).

Then, potassium 3-(2-isopropyl-1,2-dicarba-*closo*-dodecaboran-1-yl)-3-phenylpropanoate was obtained as follows [32]: in a 50 mL round-bottom flask equipped with a stir bar, 0.4 mmol of 3-(2-isopropyl-1,2-dicarba-*closo*-dodecaboran-1-yl)-3-phenylpropanoic acid and 0.4 + 10% mmol of potassium were added, along with 10 mL of THF. The reaction mixture was stirred for three days until all the metal reacted and precipitated as a white powder. The precipitate was then filtered using a Schott glass filter and washed with THF.

FTIR spectrum, ν, cm^−1^: 3200–3600 (O-H, H_2_O), 3050 (C-H, ar.), 2985–2985 (C-H), 2570, 2618 (B-H), 1575 (COO^−^, ν_asym), 1453 (C=C, ring modes), 1390 (COO^−^, ν_sym), 1082 (in-plane, C-H), 705 (out-of-plane, C-H).

^1^H NMR (500 MHz, DMSO-d6): δ = 1.32–2.41 (m, 10H, B-H), 1.23 (dd, J = 12.0, 5.5 Hz, 6H, CH(CH_3_)_2_), 2.48–2.44 (m, 2H, -CH_2_-), 2.97 (td, J = 13.5, 6.7 Hz, 1H, CH(CH_3_)_2_), 3.37 (s, H_2_O), 4.05 (m, 1H, C-H), 7.23–7.18 (m, 2H, Csp_2_H), 7.35–7.26 (m, 3H, Csp_2_H).

^13^C NMR (20 MHz, D_2_O): δ = 24.23 (-CH_3_), 24.51 (-CH_3_), 25.29 (C-H), 44.67 (C-H), 67.98 (-CH_2_-), 87.45 (C_4_, carborane core), 90.25 (C_4_, carborane core), 128.49 (Csp_2_H, 5 atoms), 140.19 (C_4_, phenyl), 178.35 (C_4_, -COO^−^).

^11^B NMR (26 MHz, D_2_O): δ = −5.24 (m, 10B).

### 2.6. Precipitation of Carborane Compound onto Fe_3_O_4_@Au-APTES Nanoparticles

Functionalized Fe_3_O_4_@Au-APTES nanoparticles were combined with the carborane compound in deionized water and subjected to ultrasonic treatment for 10 min. Ultrasonic treatment was applied to ensure uniform dispersion of the nanoparticles. While maintaining continuous mechanical stirring, hydrochloric acid was added dropwise until the pH reached 3. The reaction mixture was then stirred for an additional 24 h. The resulting nanoparticles were then washed with deionized water and dried at room temperature. At pH 3, the amino groups introduced by APTES become protonated, enabling electrostatic interaction with the carborane compound (which is negatively charged). Additionally, under acidic conditions, the potassium salt of the carborane-containing β-aryl aliphatic acid is hydrolyzed.

### 2.7. Carborane Release from NPs

**Figure 1:**
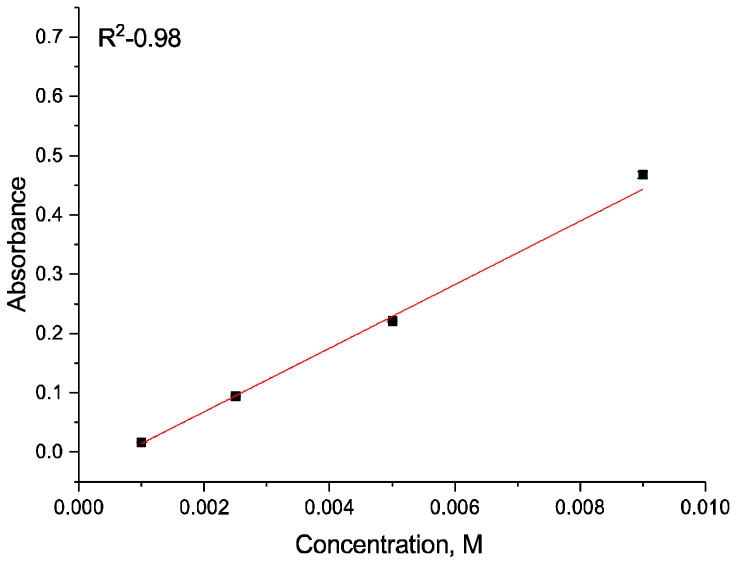
Calibration curve of carborane concentration in PBS solution.

A single PBS tablet (Sigma-Aldrich, Merck KGaA, Darmstadt, Germany) was dissolved in 100 mL of deionized water. To 15 mL of this PBS solution, 0.1 g of Fe_3_O_4_@Au–APTES–carborane nanoparticles was added. The suspension was incubated on a shaker at 36.6 °C. Aliquots were collected at 5 and 30 min and subsequently at 1 and 24 h. Thereafter, samples were taken every 24 h for one week, with the final measurement performed 316 h from the start of the experiment. Carborane concentration was quantified by UV–visible spectroscopy at 260 nm using a previously established calibration curve (Figure 1).

### 2.8. Methods of Characterization

Fourier-transform infrared (FTIR) spectra were acquired using an InfraLUM FT-08 spectrometer (Lumex Instruments, Saint Petersburg, Russia) equipped with an ATR accessory, operating over a spectral range of 400–4000 cm^−1^. Each spectrum was collected over 25 scans with a resolution of 2 cm^−1^.

Specific surface area, pore volume, and pore diameter were evaluated via Brunauer–Emmett–Teller (BET) analysis using He/N_2_ gas adsorption on a V-Sorb 2800P surface area analyzer (Gold APP Instruments Corp., Ltd., Hong Kong, China). Before measurement, samples were degassed at 120 °C.

SEM images were obtained using a SU8020 scanning electron microscope (Hitachi, Tokyo, Japan).

Transmission electron microscopy (TEM) was performed using a JEM-2800 electron microscope operating at 280 kV (JEOL, Tokyo, Japan).

X-ray diffraction (XRD) was conducted at ambient temperature using a SmartLab diffractometer (Rigaku Corporation, Tokyo, Japan) equipped with a Cu-Kα radiation source (λ = 1.5406 Å). Diffraction data were processed and analyzed using DIFFRAC.EVA software (version 4.2.1; Bruker AXS GmbH, Karlsruhe, Germany).

Dynamic light scattering (DLS) measurements were carried out in phosphate-buffered saline (PBS) at 36.6 °C using a NanoBrook 90Plus Zeta particle size analyzer (Brookhaven Instruments, Holtsville, NY, USA) following a 5 min equilibration period. Zeta potential was determined under identical temperature conditions via phase analysis light scattering (electrophoretic light scattering, ELS), with calculations based on the Smoluchowski approximation. The PBS pH was carefully adjusted using phosphoric acid and sodium hydroxide to ensure accurate readings.

The magnetic properties were analyzed using a vibrating sample magnetometer (VSM) integrated into a Liquid Helium Free High Field Measurement System (Cryogenic Ltd., London, UK). The measurements were conducted using the induction method, which detects the electromotive force induced in the signal coils by a magnetized sample oscillating at a fixed frequency under an applied magnetic field of ±1 T at 300 K.

### 2.9. Preparation of Stock Solution and Elemental Analysis

A stock solution was prepared by dispersing approximately 10 mg of nanoparticle powder in 5 mL of physiological saline, followed by sonication at 20 kHz using a 130 W VibraCell™ VCX 130PB ultrasonic processor (Sonics & Materials, Inc., Newton, CT, USA) until a uniform suspension was achieved. Between experiments, the stock solution was stored at −20 °C. The elemental concentrations of Fe, B, and Au were quantified using inductively coupled plasma atomic emission spectroscopy (ICP-AES) with a Shimadzu ICPS-8100 Twin Sequential ICP Emission Spectrometer (Shimadzu, Inc., Tokyo, Japan). For analysis, at least three aliquots of 50 µL were transferred into heat-resistant polypropylene tubes. To each, 3 mL of freshly prepared aqua regia (HNO_3_:HCl, 1:3, *v*/*v*) was added, and the mixtures were heated at 115 °C for at least 2 h. Following digestion, the samples were brought to a final volume of 10 mL using Milli-Q water. Upon acid dilution, a white precipitate formed, later confirmed to be carborane. To assess elemental concentrations in the soluble and insoluble fractions, samples were subjected to centrifugation at 30,000 rpm for 5 min using a Himac CT6E centrifuge (Hitachi, Tokyo, Japan). The supernatant and sediment were separated. The sediment was resuspended in 100 µL of DMSO, and complete dissolution into a transparent solution was confirmed. Milli-Q water was added to the solution to adjust the final volume to 10 mL. Both the acidic supernatant (enriched in Fe and Au) and the neutral fraction (resuspended precipitate, primarily containing B) were passed through 0.20 µm syringe filters before ICP-AES analysis. To reduce spectral interference, the following emission wavelengths were selected: 259.940 nm for Fe, 208.959 nm for B, and 242.795 nm for Au. Elemental concentrations were reported in ppm based on the mean of three independent measurements per sample.

### 2.10. Human Glioblastoma Cell Line

U251MG cells were procured from the American Type Culture Collection (ATCC, Manassas, VA, USA) and cultured at 37 °C in a humidified incubator with 5% CO_2_. Cells were maintained in Dulbecco’s Modified Eagle’s Medium (DMEM; Sigma-Aldrich, Catalog No. D5796, St. Louis, MO, USA) containing 4500 mg/L glucose, L-glutamine, and sodium bicarbonate. The medium was supplemented with 10% fetal bovine serum (HyClone™, GE Healthcare Life Sciences, Catalog No. SH30396.03, South Logan, UT, USA) and 1% penicillin-streptomycin (10,000 U/mL penicillin and 10 mg/mL streptomycin (Sigma-Aldrich, Catalog No. P0781).

### 2.11. Cytotoxicity Evaluation

The potential cytotoxicity of the nanoparticles was examined using the MTS assay, following established methodologies [31,33]. Briefly, U251MG cells were seeded into 96-well plates at a density of 4 × 10^4^ cells per well in 100 µL of complete medium and incubated for 24 h. After this period, the medium was replaced with fresh medium containing nanoparticles at concentrations ranging from 0 to 500 µg Fe/mL, followed by another 24 h incubation period. The nanoparticle-containing medium was then discarded, and the wells were gently washed twice with PBS. Subsequently, 100 µL of a solution composed of one part MTS reagent (3-(4,5-dimethylthiazol-2-yl)-5-(3-carboxymethoxyphenyl)-2-(4-sulfophenyl)-2H-tetrazolium) combined with phenazine methosulfate (PMS) (CellTiter 96^®^ AQueous One Solution, Promega Corporation, Madison, WI, USA) and five parts of fresh medium was added to each well. The plates were incubated for an additional 2 h. To eliminate interference from nanoparticle absorbance, the MTS-containing supernatant was transferred to clean, particle-free plates before absorbance measurement. Optical density at 490 nm was measured using a microplate reader (Berthold Technologies GmbH & Co. KG, Bad Wildbad, Germany), and cell viability was calculated as a percentage relative to untreated controls.

### 2.12. Accumulation of Fe, B, and Au in Tumor Cells

Intracellular levels of Fe, B, and Au were quantified by inductively coupled plasma atomic emission spectroscopy (ICP-AES) using the same analytical procedures previously applied to the stock solution elemental analysis [31,33,34]. U251MG cells (1 × 10^6^) were seeded in 25 cm^2^ culture flasks containing 3 mL of DMEM and incubated for 24 h at 37 °C in a humidified atmosphere with 5% CO_2_. The medium was subsequently replaced with DMEM supplemented with nanoparticles at a concentration corresponding to 150 ppm Fe, followed by a further 24 h incubation period. After treatment, cells were washed twice with PBS to remove unbound nanoparticles, detached using L-Trypsin-EDTA (2.5 g/L trypsin, 1 mmol/L EDTA; Nacalai Tesque, Inc., Kyoto, Japan), and transferred into polypropylene tubes. The cells were then pelleted by centrifugation, the supernatant was removed, and 3 mL of freshly prepared Aqua Regia (1:3 ratio of concentrated HNO_3_ to HCl) was added. The samples were heated at 115 °C for a minimum of 4 h. Following cooling, the digests were diluted with Milli-Q water to a final volume of 5 mL, at which point a white precipitate was observed. The white precipitate was presumed to be undissolved carborane. Samples were transferred to conical tubes and centrifuged at 30,000 rpm for 5 min to separate the soluble and insoluble components. The supernatant, primarily containing Fe and Au, was carefully collected. The pellet, believed to contain carborane, was resuspended in 100 µL of DMSO, resulting in a visually confirmed clear solution. This mixture was then diluted with Milli-Q water to a final volume of 5 mL. Both the supernatant (acidic fraction) and the resuspended pellet (neutral fraction, primarily containing B) were passed through 0.20 µm syringe filters and analyzed by ICP-AES.

## 3. Results and Discussion

**Figure 2:**
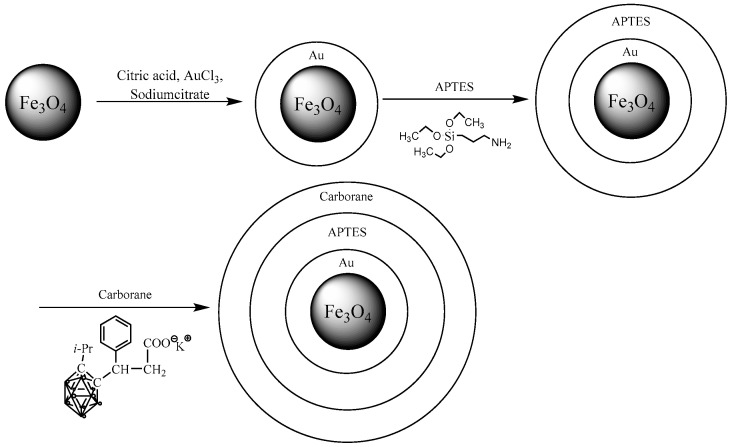
Scheme of Fe_3_O_4_ modification.

A schematic representation of Fe_3_O_4_-NP modification is shown in Figure 2.

**Figure 3:**
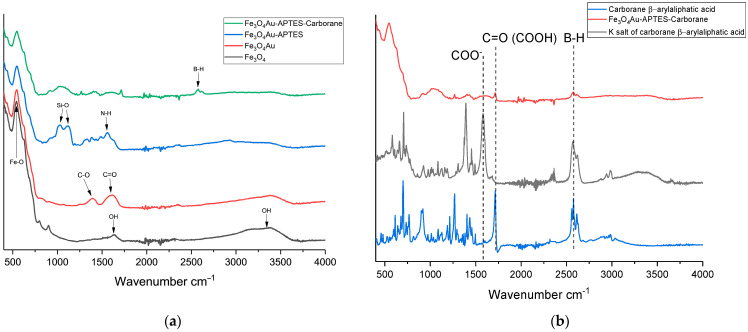
FTIR spectra of Fe_3_O_4_ at different stages of modifications (**a**); FTIR spectrum dissociation of potassium salt of carborane containing β-aryl aliphatic acid (**b**).

Figure 3a shows the FTIR spectra corresponding to the successive modifications of Fe_3_O_4_ nanoparticles. The FTIR spectra of the initial Fe_3_O_4_ nanoparticles reveal characteristic peaks corresponding to Fe-O bonds at 580 cm^−1^, as well as OH vibrations at 1630 cm^−1^ and 3405 cm^−1^. Upon modification with gold to form a “core–shell” structure, as described by Fadeev et al. (2020), vibrations of C=O groups from sodium citrate appear at 1730 cm^−1^ [29]. Subsequent functionalization of Fe_3_O_4_@Au nanoparticles with APTES is indicated by peaks corresponding to Si-O-Si bonds at 1012 cm^−1^ and 1296 cm^−1^, along with the N-H bond at 1568 cm^−1^. When a carborane compound (the potassium salt of a carborane-containing β-aryl aliphatic acid) is deposited onto amino groups pre-protonated with hydrochloric acid (pH 3), the FTIR spectrum shows a characteristic peak at 2600 cm^−1^. Additionally, the dissociation of the potassium salt is evident from the reappearance of the COOH bond, which is characteristic of β-aryl aliphatic acid (Figure 3b).

**Figure 4:**
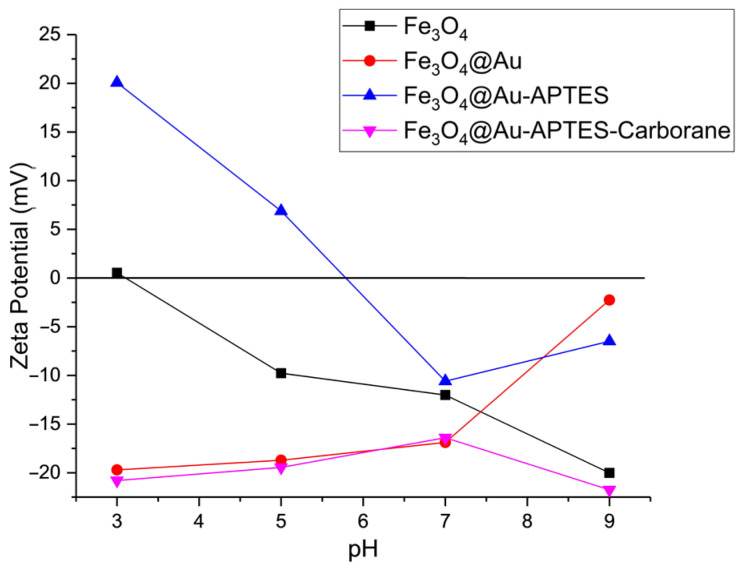
Zeta potential of nanoparticles at different stages of modification.

Zeta potential data for the synthesized nanoparticles are shown in Figure 4. Measurements were conducted in electrophoretic light scattering (ELS) mode, applying Smoluchowski’s approximation, in PBS solutions with varying pH levels. The pH was adjusted using phosphoric acid and sodium hydroxide. The isoelectric point of the initial Fe_3_O_4_ nanoparticles was observed at pH 3, which is typical. Within the pH range of 5–9, the formation of Fe–O^−^ surface groups contributes to the development of a negative zeta potential. Modifying Fe_3_O_4_ nanoparticles with gold shifts the isoelectric point toward a more alkaline pH, likely due to the presence of unreacted citric acid groups.

Further modification with APTES ((3-aminopropyl)triethoxysilane) causes the zeta potential to shift to +20 mV in an acidic environment, indicating a positive surface charge attributed to NH_2_ groups. Following the addition of carborane compounds, the zeta potential stabilizes at −20 mV, suggesting that the amino groups are fully coated by the carborane layer, resulting in a negative surface charge.

**Figure 5:**
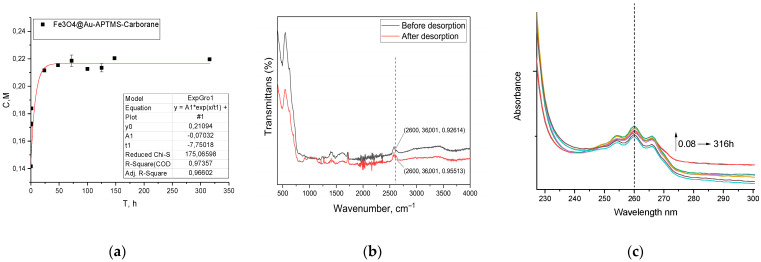
The stability of carborane on NPs in a phosphate-buffered saline (PBS) solution (**a**) and FTIR analysis before and after desorption (**b**); UV-vis spectra of the solution during carborane release (**c**).

For effective BNCT, it is crucial that the boron-containing compound remains within the tumor cell in association with the nanoparticle and is not released into the extracellular medium. As shown in Figure 5a, only a minor fraction of the carborane is released in PBS during the initial 48 h (Figure 5a). However, FTIR spectroscopy (Figure 5b) analysis confirms that the majority of the carborane remains bound to the nanoparticles and is retained for over 316 h, demonstrating the stability of the system under these conditions. Although PBS mimics the ionic strength and pH of blood, it does not fully replicate the complexity of biological fluids. Therefore, the observed stability in PBS should be considered a preliminary indicator.

Table 1 summarizes the surface area and pore structure characteristics of the nanoparticle samples, as measured using He/N_2_ adsorption (adsorption–desorption isotherms for the samples are presented in Figure S1). Pristine Fe_3_O_4_ nanoparticles exhibited a specific surface area of 102.16 m^2^/g, a total pore volume of 0.34 cm^3^/g, and an average pore diameter of 13.31 nm. Following gold coating to form a core–shell Fe_3_O_4_@Au structure, the specific surface area slightly decreased to 96.09 m^2^/g, while the total pore volume increased to 0.46 cm^3^/g, likely due to the enhanced morphological complexity introduced by the gold layer. Functionalization with APTES further increased both the surface area and pore volume, reaching 108.69 m^2^/g and 0.53 cm^3^/g, respectively, consistent with the porous and branched structure of the silane coating. However, subsequent conjugation with the boron-containing carborane compound led to a reduction in these values, with the specific surface area dropping to 94.69 m^2^/g and the pore volume to 0.51 cm^3^/g, presumably due to partial pore occupancy by carborane molecules.

The elemental content of the nanoparticles was determined by the EDX method (Table 2). After creating a core–shell structure with gold, carbon (21.5%) and gold (5.3%) atoms were detected. The next modification of APTES led to the detection of nitrogen (3.6%) and silicon (1.2%) atoms. The final nanoparticles coated with carborane showed boron atoms (28.9%); however, it should be noted that EDX for light elements may not be determined correctly due to overlapping peaks of carbon and other light elements. Therefore, the concentration was also determined by analytical ICP-AES during biological studies (the results are presented below).

**Figure 6:**
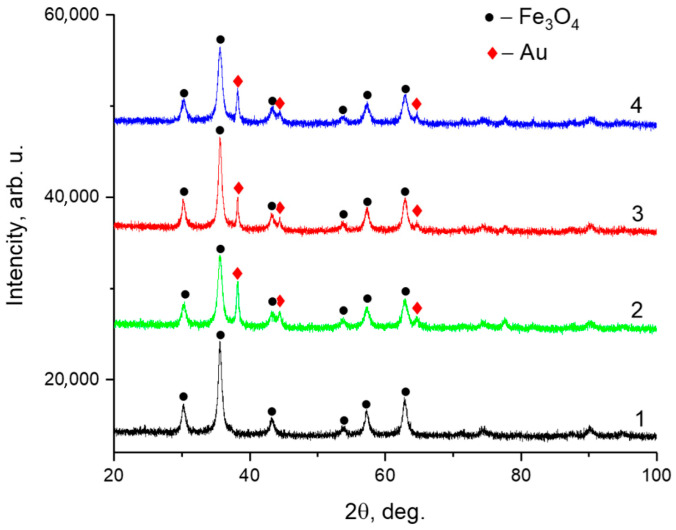
X-ray diffraction patterns of the obtained samples: (1) Fe_3_O_4_, (2) Fe_3_O_4_@Au, (3) Fe_3_O_4_@Au–APTES, and (4) Fe_3_O_4_@Au–APTES–carborane.

The phase composition and crystalline structure of the samples were analyzed by powder XRD performed using a Rigaku SmartLab diffractometer operating in Bragg–Brentano geometry with Cu Kα radiation (Figure 6). The diffraction patterns confirmed the presence of Fe_3_O_4_ (magnetite), corresponding to the cubic spinel structure with space group *Fd-3m*. Additionally, characteristic peaks of metallic gold (*Au*) were observed, associated with a face-centered cubic structure (space group *Fm-3m*). The calculated lattice parameters and average crystallite sizes are summarized in Table 3.

**Figure 7:**
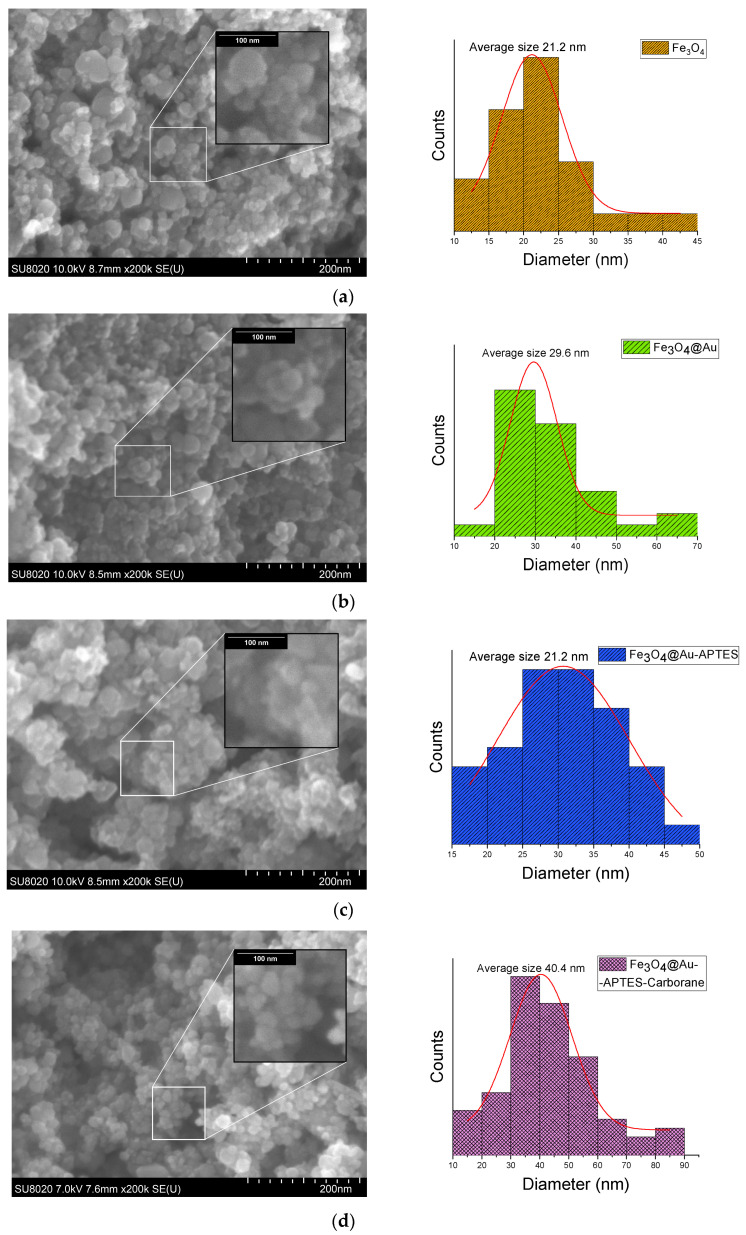
SEM images and size distribution histograms of (**a**) Fe_3_O_4_, (**b**) Fe_3_O_4_@Au, (**c**) Fe_3_O_4_@Au–APTES, and (**d**) Fe_3_O_4_@Au–APTES–carborane nanoparticles.

SEM images of Fe_3_O_4_ nanoparticles at various stages of modification, including Fe_3_O_4,_ Fe_3_O_4_@Au, Fe_3_O_4_@Au-APTES, and Fe_3_O_4_@Au–APTES–carborane, reveal a gradual increase in nanoparticle size (Figure 7). The average size of Fe_3_O_4_@Au increased from 29 nm to 30 nm after APTES functionalization, and it reached 40 nm with the incorporation of the carborane compound.

DLS analysis demonstrated a consistent increase in the hydrodynamic diameter of Fe_3_O_4_ at all stages of modification (Table 4). Pure Fe_3_O_4_ had an average size of 156 nm with a diffusion coefficient of 3.144 × 10^−8^ cm^2^/s. Gold coating increased the size to 506 nm and decreased diffusion to 9.777 × 10^−9^ cm^2^/s, confirming the deposition of Au nanoparticles. The final modification with APTES and conjugation with carborane led to a further increase in the diameter to 689 nm and a decrease in the diffusion coefficient to 7.317 × 10^−9^ cm^2^/s, while the polydispersity remained in the range of 0.186–0.211. Both DLS and SEM analyses show a similar trend of an increasing nanoparticle size with each modification, but with different absolute values. This discrepancy is explained by differences in measurement methods: SEM records the size of solid particles, while DLS takes into account the hydrodynamic diameter, including the hydration shell and possible aggregation.

**Figure 8:**
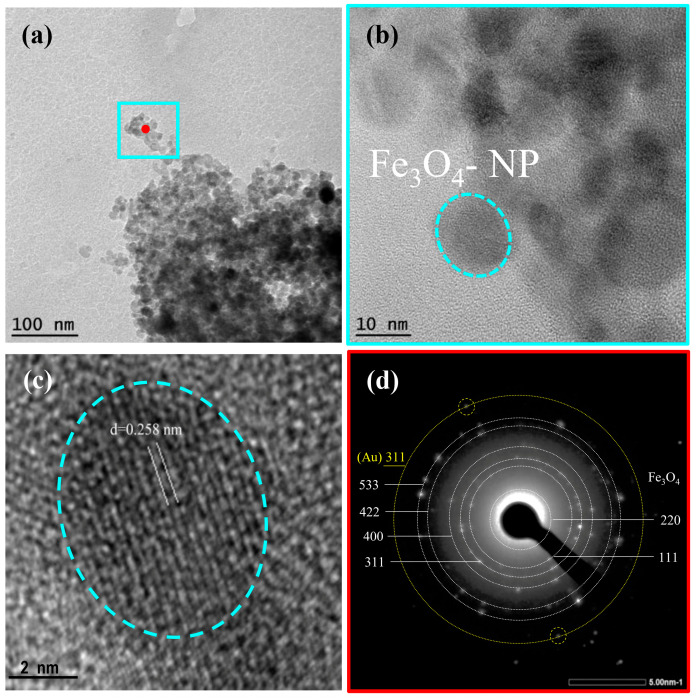
Material and structural characterization of Fe_3_O_4_@Au–APTES–carborane particles. (**a**) Low-resolution transmission electron microscopy (TEM) image of Fe_3_O_4_@Au–APTES–carborane particles. (**b**,**c**) High-resolution TEM images of Fe_3_O_4_@Au–APTES–carborane particles showing their morphology and surface structure. (**d**) Selected-area electron diffraction (SAED) pattern obtained from the region shown in (**a**), where yellow corresponds to Au and white corresponds to Fe_3_O_4_ lattice orientation.

TEM analysis results of Fe_3_O_4_@Au-APTES–carborane nanoparticles are presented in Figure 8. The low-magnification TEM image (Figure 8a) reveals aggregated nanoparticles with individual Fe_3_O_4_ domains ranging from 10 to 20 nm in size. This is in good agreement with the crystallite size estimated from XRD, which confirms the formation of nanocrystalline magnetite. High-resolution TEM images (Figure 8b,c) display well-defined lattice fringes, with an interplanar spacing of approximately 0.258 nm (Figure 8c), corresponding to the (311) plane of Fe_3_O_4_. The selected-area electron diffraction (SAED) pattern (Figure 8d) shows distinct concentric rings with bright diffraction spots, confirming the polycrystalline nature of the sample. Since gold is deposited as a thin layer on the surface of the iron oxide cores, its individual crystal lattice may not be clearly visualized in high-resolution TEM images. However, a distinct ring corresponding to the (311) plane of gold is clearly visible in the SAED pattern (Figure 8d), supporting the presence of the gold shell.

Compared to the SEM results, which show an average particle size of ~40 nm, the smaller sizes observed in TEM suggest that SEM reflects aggregated structures, including surface coatings with Au, APTES, and carborane. In contrast, TEM and XRD provide insights into the size of the crystalline magnetite cores. The organic layers (APTES and carborane), due to their low electron contrast, are not visible in TEM but were confirmed by complementary techniques (FTIR, zeta potential, ICP-AES).

TEM elemental maps (Figure S2) confirm the presence of Fe and O as the primary components of the nanoparticle cores. Au is also detected across the sample, indicating successful deposition. The presence of B, N, Si, and C further supports successful surface functionalization with APTES and the carborane compound.

As mentioned earlier, the magnetic properties of the composites were investigated using VSM integrated into the universal measurement system (automated vibrating magnetometer) under an applied magnetic field of ±1 T at 300 K. Figure S3 shows the hysteresis loops of Fe_3_O_4_ and the final Fe_3_O_4_@Au–APTES–carborane nanoparticles. The calculated magnetic parameters were as follows: Fe_3_O_4_ exhibited a coercivity of 14 Oe, a saturation magnetization of 62.1 emu/g, and a remanent magnetization of 1.21 emu/g. These are typical values for Fe_3_O_4_. The Fe_3_O_4_@Au–APTES–carborane nanoparticles showed a coercivity of 9.4 Oe, a saturation magnetization of 57.2 emu/g, and a remanent magnetization of 1.16 emu/g.

The properties of the modified particles differ from those of the original ones due to changes in the magnetic phase composition, as evidenced by a reduction in saturation magnetization and a decrease in coercivity, indicating alterations in the magnetic core. The surface coating on the particles increases the interparticle spacing, which in turn leads to a decrease in remanent magnetization.

**Figure 9:**
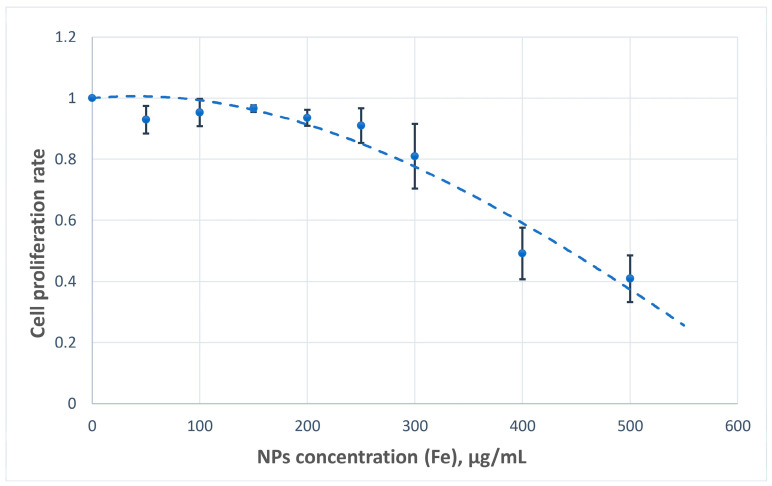
Proliferation of U251MG cells after incubation with nanoparticles.

The cytotoxic potential of the nanoparticles was assessed based on their impact on U251MG cell proliferation. Across the tested concentration range, the nanoparticles demonstrated acceptable biocompatibility, with minimal cytotoxic effects observed at concentrations of up to 300 µg Fe/mL (Figure 9).

Nanoparticles exhibited acceptable cytocompatibility, with minimal inhibition of cell proliferation observed at 0 to 300 µg Fe/mL in the medium. These findings support the feasibility of advancing to in vivo studies, as the effective therapeutic boron concentrations reported in tumor tissues typically range between 20 and 30 µg/mL.

These findings are also consistent with our previous cytotoxicity assessments reported for similar nanoplatforms. In particular, in our earlier work published in [35], Fe_3_O_4_-aminated nanoparticles with APTMS exhibited an IC_50_ value of 0.091 mg/mL, whereas Fe_3_O_4_-aminated-carborane nanoparticles showed a markedly reduced cytotoxicity with an IC_50_ value of 0.405 mg/mL. This reduction was attributed to the neutralization of surface amino groups upon carborane conjugation, which decreased electrostatic interactions with negatively charged cell membranes. Fe_3_O_4_@Au nanoparticles have been shown to possess even lower intrinsic toxicity in other studies; for example, they exhibited negligible cytotoxicity in HEK293 cells across a wide range of concentrations [36]. Furthermore, the carborane compound (potassium 3-(2-isopropyl-1,2-dicarba-closo-dodecaboran-1-yl)-3-phenylpropanoate) itself was previously characterized in [32], where it demonstrated relatively high cytotoxicity against HDFn fibroblasts and MCF-7 cells (IC_50_ = 20.1 and 25.0 µg/mL, respectively). Together, these results confirm that immobilizing carborane on the nanoparticle surface reduces its inherent toxicity while preserving its boron delivery capacity.

Efficient accumulation of nanoparticles with their active elements in the cells moves us closer to the possibility of further experiments on cell and animal irradiation. Although iron is the main component of the nanoparticles, measurable intracellular levels of boron and gold confirmed the structural integrity of the particles following cellular uptake. To more accurately assess nanoparticle accumulation, elemental concentrations were calculated per 10^6^ cells rather than by mass, as the latter may be affected by residual medium and the low biomass of cultured cells. The number of accumulated atoms in a cell was determined using the following equation:

$$N=\left(\frac{C}{m}\times N_{A}\right)/10^{6},$$

where N represents the number of atoms in a cell, C represents the concentration of the analyzed element in one million (10^6^) cells (in grams), m represents the molar mass of the element (g/mol), and N_a_ represents Avogadro’s number (≈6.022 × 10^23^ mol^−1^). The calculated values are shown in Table 5.

The quantity of boron (^10^B) atoms required in a tumor cell to achieve the BNCT effect is estimated to be approximately 10^9^ atoms [37]. In this study, we found that the measured boron accumulation reached 1.742 × 10^10^ atoms per tumor cell, exceeding the established threshold by an order of magnitude. These findings confirm that the current elemental composition of the nanoparticles provides a sufficient boron dose to initiate neutron capture and potentially inhibit tumor cell proliferation.

A comparison of the obtained results with previously reported data should be made with caution as boron uptake strongly depends on the specific cell type and the physiological state of the cells. For example, in the study by Capala et al. (1996), the accumulation of boron from boronophenylalanine (BPA) and sodium borocaptate (BSH) was investigated in several cell lines, including U-343 MGa human glioma cells, B16 murine melanoma cells, bovine aortic endothelial cells, and GM498 human fibroblasts, under both confluent and non-confluent conditions [38]. The results demonstrated significant variability in boron uptake depending on the cell type and the compound used. Specifically, in U-343 MGa glioma cells, the maximum intracellular boron accumulation was approximately 4 × 10^9^ atoms/cell for BSH and 5.6 × 10^9^ atoms/cell for BPA. In B16 melanoma cells, uptake reached 1.2 × 10^10^ atoms/cell for BSH and 6 × 10^9^ atoms/cell for BPA. In bovine aortic endothelial cells, boron accumulation was 1.8 × 10^10^ atoms/cell for BSH and 7 × 10^9^ atoms/cell for BPA. In GM498 human fibroblasts, the boron content varied depending on confluency: in non-confluent cultures, values of 1 × 10^10^ atoms/cell for BSH and 5 × 10^9^ atoms/cell for BPA were reported, while in confluent cultures, the values decreased to 3 × 10^9^ atoms/cell for BSH and 4 × 10^9^ atoms/cell for BPA. Thus, the developed Fe_3_O_4_@Au–APTES–carborane nanoparticles demonstrate competitive boron delivery performance. Furthermore, their multifunctionality offers additional advantages, including the potential for magnetic guidance and imaging, making them a promising platform for BNCT and theranostic applications.

The optimal amount of gold required in the agent for accurate absorbed dose estimation remains an open research question. In our previous in vitro study, we demonstrated that gold can efficiently emit diagnostic gamma rays upon neutron irradiation and that the amount required is significantly lower than the quantity of boron needed to achieve therapeutic efficacy [31]. Upon neutron irradiation, ^197^Au undergoes transmutation to ^198^Au, a β^−^ emitter with a half-life of 2.7 days and potential therapeutic relevance. This process provides a synergistic effect when combined with the high-linear energy transfer (LET) α-particles generated by the ^10^B(n,α)^7^Li reaction. The incorporation of gold thus introduces an additional layer of therapeutic activity beyond boron delivery, enhancing the overall efficacy of the treatment. Based on our results, we hypothesize that the gold content in the analyzed nanoparticles may be sufficient for estimating the boron dose after irradiation. However, this hypothesis requires validation through further experimental studies.

Iron, due to its inherent magnetic properties, holds considerable promise for use in various therapeutic modalities. The field of magnetic nanoparticle systems is rapidly evolving, introducing novel agents and strategies for cancer treatment. Wang et al. developed nanoparticles polyhedral in shape with magnetic properties and CD44 antibodies attached, which induced apoptosis in gastric cancer stem cells through mechanical disruption under rotating magnetic fields [39]. Zhang et al. demonstrated enhanced tumor accumulation (more than 2.6-fold) of iron oxide–transferrin (SPIO–Tf)-based nanoparticles with superparamagnetic properties via in vivo alternating magnetic field (AMF) stimulation, presenting an effective magnetothermal targeting strategy in adenocarcinoma models that may be applicable to our nanoparticles [40]. Other researchers have employed iron oxide-based multicore nanoparticles for combination therapies and localized hyperthermia in various cancer cells [41], involving lung cancer, breast adenocarcinoma, pancreatic carcinoma, colon cancer, triple-negative breast cancer, and uveal melanoma cell lines. Xie et al. published a review on surface-engineered magnetic ferric oxide nanosized particles with drug delivery applications and diagnostic properties for breast cancer [42], while Forgham et al. synthesized fluoropolymer-engineered magnetic nanoparticles penetrating the blood–brain barrier and enabling gene silencing in medulloblastoma [43]. To emphasize their theranostic utility, Tao et al. developed magnetic nanoparticles labeled with enhanced green fluorescent protein, delivered by neural progenitor cells, which enabled both photothermal therapy and multimodal imaging for glioma treatment, including MRI and fluorescence-guided surgery [44].

In this study, we proposed and applied a new strategy of Fe_3_O_4_ nanoparticle modification by incorporating boron and gold to create a multifunctional theranostic agent. This nanoplatform is capable not only of initiating neutron capture reactions but also of emitting secondary gamma photons, enabling absorbed dose estimation. In addition, the intrinsic magnetic properties of the nanoparticles offer potential for integration into various combination therapies, including magnetic targeting and hyperthermia. Initially, the nanoparticles were synthesized using natural boron; however, for optimal therapeutic efficacy in BNCT, the ^10^B isotope is required. Following further optimization of the nanoparticle formulation and development of a delivery system with tumor targeting, natural boron will be replaced with ^10^B. Commercially available ^10^B-enriched *o*-carborane or synthesized ^10^B-labeled analogs, prepared using isotopically enriched elemental boron, can be used as starting materials. Importantly, the synthetic route for obtaining potassium 3-(2-isopropyl-1,2-dicarba-*closo*-dodecaboran-1-yl)-3-phenylpropanoate remains unchanged, making the incorporation of ^10^B both practical and scalable. This approach ensures the compatibility of the developed nanoplatform with the requirements of BNCT.

Several limitations should be acknowledged in the present study. We assessed the baseline stability of Fe_3_O_4_@Au–APTES–carborane nanoparticles using PBS, a commonly used physiologically relevant medium that mimics the pH and ionic strength of blood plasma. While PBS provides a controlled environment for initial evaluation, we acknowledge its limitation in replicating the full complexity of biological fluids. However, the fact that cellular uptake experiments were performed in DMEM supplemented with 10% FBS further supports the stability of the surface-functionalized nanoparticles in biologically relevant conditions. Notably, the nanoparticles remained in contact with serum proteins throughout incubation. Despite the potential for serum components to displace loosely bound molecules, the observed high intracellular accumulation of boron, iron, and gold, as detected by ICP-AES, strongly suggests that carborane remained stably immobilized on the nanoparticle surface. If carborane had detached due to serum interactions, it likely would have been washed away during the rinsing steps before analysis, resulting in lower boron levels in the cells. Furthermore, unlike BPA [11], carborane does not possess active tumor-targeting properties and can only enter cells via passive diffusion, which is generally inefficient. Therefore, the efficient boron delivery observed here further indicates that carborane was internalized in association with the nanoparticles rather than as free molecules. These findings collectively support the robustness of our surface modification strategy and the potential applicability of the nanoparticles in complex biological environments. Additional in vivo studies are planned to evaluate their biodistribution, pharmacokinetics, and therapeutic performance under physiologically relevant conditions.

To assess the biocompatibility of Fe_3_O_4_@Au–APTES–carborane nanoparticles, we conducted 24 h cytotoxicity studies using the U251MG glioblastoma cell line. Extended incubation times (48 or 72 h) were not evaluated, as rapid proliferation and density-dependent effects in U251MG cells could introduce variability due to nutrient depletion and accumulation of metabolic byproducts, potentially confounding the interpretation of nanoparticle-induced effects. By limiting exposure to 24 h, we ensured consistent conditions across wells and a reliable assessment of nanoparticle-associated cytotoxicity. To further validate these findings, additional cytotoxicity experiments were performed in primary mouse astrocyte (AWT) cells, which demonstrated even higher cell viability across comparable nanoparticle concentrations (Figure S4). These results confirm the good biocompatibility of the developed nanoplatform and support its suitability for further biological applications. As cytotoxicity assessment is a critical step in the development of BNCT agents, these findings provide a strong foundation for future in vivo studies aimed at evaluating therapeutic efficacy and safety under more physiologically relevant conditions.

Although effective boron accumulation was achieved, further enhancement of boron incorporation into the nanoparticles may be required to ensure sufficient tumor accumulation in vivo as the results obtained in cultured tumor cells may not fully translate to animal models. Regarding boron quantification, we implemented a customized protocol to dissolve the carborane-containing sediment, as standard digestion with Aqua Regia alone did not appear to achieve complete ionization of the boron species. While our adapted approach enabled reliable measurement, further methodological refinement and validation are needed. Future studies should explore alternative or complementary digestion protocols to improve the accuracy and standardization of elemental analysis for such complex nanoparticle formulations.

## 4. Conclusions

We developed a novel strategy for modifying Fe_3_O_4_ nanoparticles by incorporating boron and gold, aiming to create multifunctional agents for cancer diagnosis and BNCT. Comprehensive characterization of the resulting nanostructures was performed using EDX, SEM, TEM, XRD, DLS, ICP-AES, and FTIR spectroscopy. The size of the final nanoparticles was 40.4 nm on average as determined by SEM. Release studies indicated that the majority of carborane remained stably associated with the nanoparticle surface. The ζ-potential stabilized at –20 mV, suggesting effective surface coating with the carborane layer. The nanoparticles demonstrated low cytotoxicity, even at concentrations exceeding therapeutic levels, and showed efficient accumulation in tumor cells at levels compatible with BNCT requirements. Future research will focus on enhancing the boron loading, improving tumor-targeting efficiency, and validating both therapeutic and diagnostic capabilities in vivo.

## Figures and Tables

**Table 1:** Data on BET surface area, pore volume, and pore width of samples.

Sample	BET Surface Area, m^2^/g	Total Pore Volume, cm^3^/g	Average Pore Width, nm
Fe_3_O_4_	102.16 ± 9.62	0.34	13.31
Fe_3_O_4_@Au	96.09 ± 8.10	0.46	18.96
Fe_3_O_4_@Au-APTES	108.69 ± 10.21	0.53	19.60
Fe_3_O_4_@Au–APTES–carborane	94.69 ± 8.51	0.51	21.34

**Table 2:** Elemental content of NPs according to EDX.

Sample	Elemental Content, %						
	Fe	O	C	Au	N	Si	B
Fe_3_O_4_	38.6	61.4	-	-	-	-	-
Fe_3_O_4_@Au	20.9	52.3	21.5	5.3	-	-	-
Fe_3_O_4_@Au-APTES	20.4	49.5	20.8	3.7	4.4	1.2	-
Fe_3_O_4_@Au–APTES–carborane	15.2	34.6	16.8	0.5	3.7	0.3	28.9

**Table 3:** XRD parameters.

№	A, Å	D, nm
Fe_3_O_4_	8.3626	10.2 ± 0.8
Fe_3_O_4_@Au	8.3656	10.0 ± 1.0
Fe_3_O_4_@Au-APTES	8.3628	9.7 ± 2.0
Fe_3_O_4_@Au–APTES–carborane	8.3644	9.4 ± 2.2

**Table 4:** DLS measurement results.

Sample	Eff. Diam. (nm)	Polydispersity	Baseline Index	Count Rate (kcps)	Data Retained (%)	Diffusion Coeff. (cm^2^/s)
Fe_3_O_4_	156 ± 2	0.186 ± 0.012	8.5 ± 1.2	447.8 ± 2.7	99.51 ± 0.54	3.144 × 10^−8^ ± 3.879 × 10^−10^
Fe_3_O_4_@Au	506 ± 47	0.198 ± 0.021	4.9 ± 3.1	402.0 ± 8.8	99.79 ± 0.46	9.777 × 10^−9^ ± 9.147 × 10^−10^
Fe_3_O_4_@Au APTES	354 ± 26	0.201 ± 0.029	5.9 ± 2.4	11.0 ± 0.2	98.81 ± 1.29	1.392 × 10^−8^ ± 1.027 × 10^−9^
Fe_3_O_4_@Au APTES–carborane	689 ± 123	0.211 ± 0.026	7.8 ± 1.3	470.2 ± 3.2	100.00 ± 0.00	7.317 × 10^−9^ ± 1.399 × 10^−9^

**Table 5:** The concentrations of Fe, B, and Au in a single U251MG cell following 24 h of incubation in the medium with nanoparticles (150 µg Fe/mL).

Elements	Amount in 10^6^ Cells, μg	Number of Atoms in A Cell
Fe	243.656	2.627 × 10^12^
B	1.298	1.742 × 10^10^
Au	5.966	1.823 × 10^10^

## Data Availability

All datasets analyzed or generated in the course of this research are accessible from the corresponding authors upon reasonable request.

## Supplementary Materials

The following supporting information can be downloaded at: https://www.mdpi.com/article/10.3390/nano15161243/s1, Figure S1: Adsorption-desorption isotherms; Figure S2: STEM–EDS analysis; Figure S3: Hysteresis loops of the samples; Figure S4: Proliferation of primary mouse astrocyte (AWT) cells after 24-h incubation with Fe_3_O_4_@Au–APTES–carborane nanoparticles.
