# Supplementary material for: Carborane-Containing Iron Oxide@Gold Nanoparticles for Potential Application in Neutron Capture Therapy
Source: Nanomaterials (Basel). 2025 Aug 13;15(16):1243. doi: 10.3390/nano15161243 (PMC12388822; doi:10.3390/nano15161243)
Supplement: Supplementary file 1 [file nanomaterials-15-01243-s001.zip › nanomaterials-3775804-supplementary.pdf]

Supplementary materials

Carborane-Containing Iron Oxide@Gold Nanoparticles for Potential Application in Neutron Capture Therapy

Zhangali A. Bekbol [1,2], Kairat A. Izbasar [*1,2], Alexander Zaboronok [1,3]*, Lana I. Lissovskaya [1,2], Haolan Yang [3], Yuriy Pihosh [4], Eiichi Ishikawa [3], Rafael I. Shakirzyanov [2], Ilya V. Korolkov [1,2]*

[1] The Institute of Nuclear Physics, Ibragimov Str. 1, 050032 Almaty, Kazakhstan; zhangali.bekbol@mail.ru (Z.A.B.); ms.defrance@mail.ru (L.I.L.); i.korolkov@inp.kz (I.V.K.)
[2] Engineering Profile Laboratory, L.N. Gumilyov Eurasian National University, Satpaev str. 5, 010008, Astana, Kazakhstan; shakirzyanov_ri@enu.kz (R.I.S.)
[3] Department of Neurosurgery, Institute of Medicine, University of Tsukuba, 1-1-1 Tennodai, Tsukuba 305-8575, Japan; s2430438@u.tsukuba.ac.jp (H.Y.); e-ishikawa@md.tsukuba.ac.jp (E.I.)
[4] Office of University Professors, The University of Tokyo, 2-11-16 Yayoi, Bunkyo-ku, Tokyo 113-8656, Japan; pihosh_y@arpchem.t.u-tokyo.ac.jp (Y.P.)
* Correspondence: a.zaboronok@md.tsukuba.ac.jp (A.Z.), kai.aslan.72@mail.ru (K.A.I.)

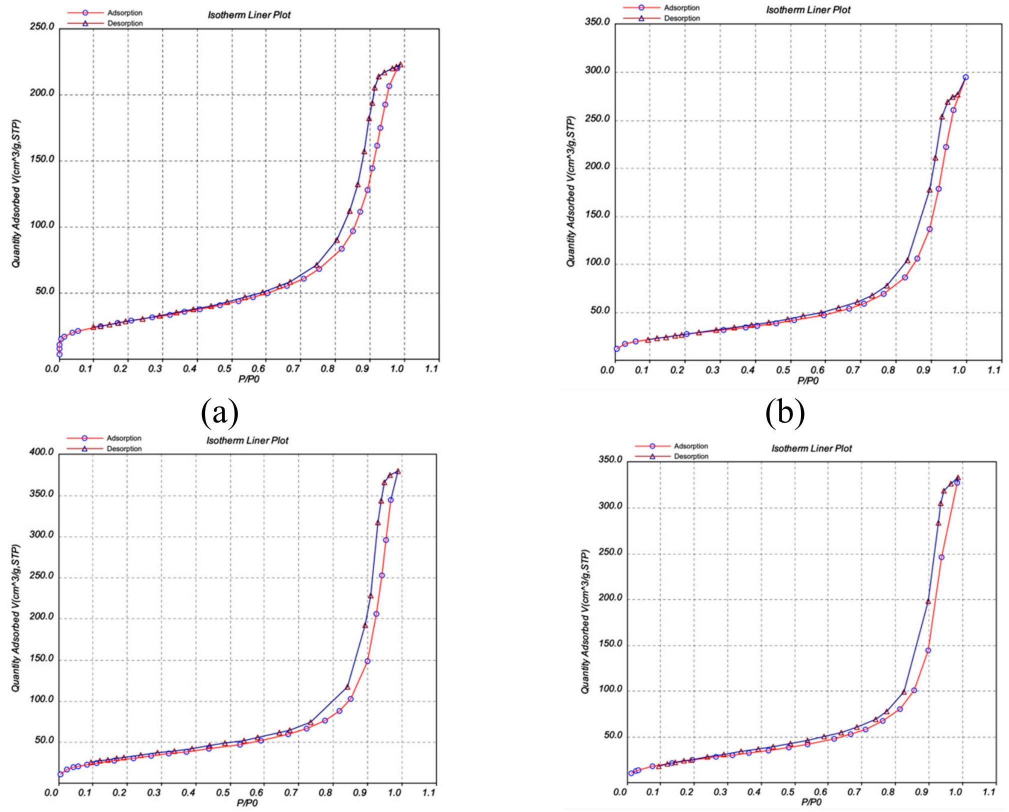

(a) (b) (c) (d)

Figure S1 - Adsorption-desorption isotherms for samples Fe_3O_4 (a), Fe_3O_4@Au (b), Fe_3O_4@Au-APTES (c) and Fe_3O_4@Au-APTES-carborane (d)

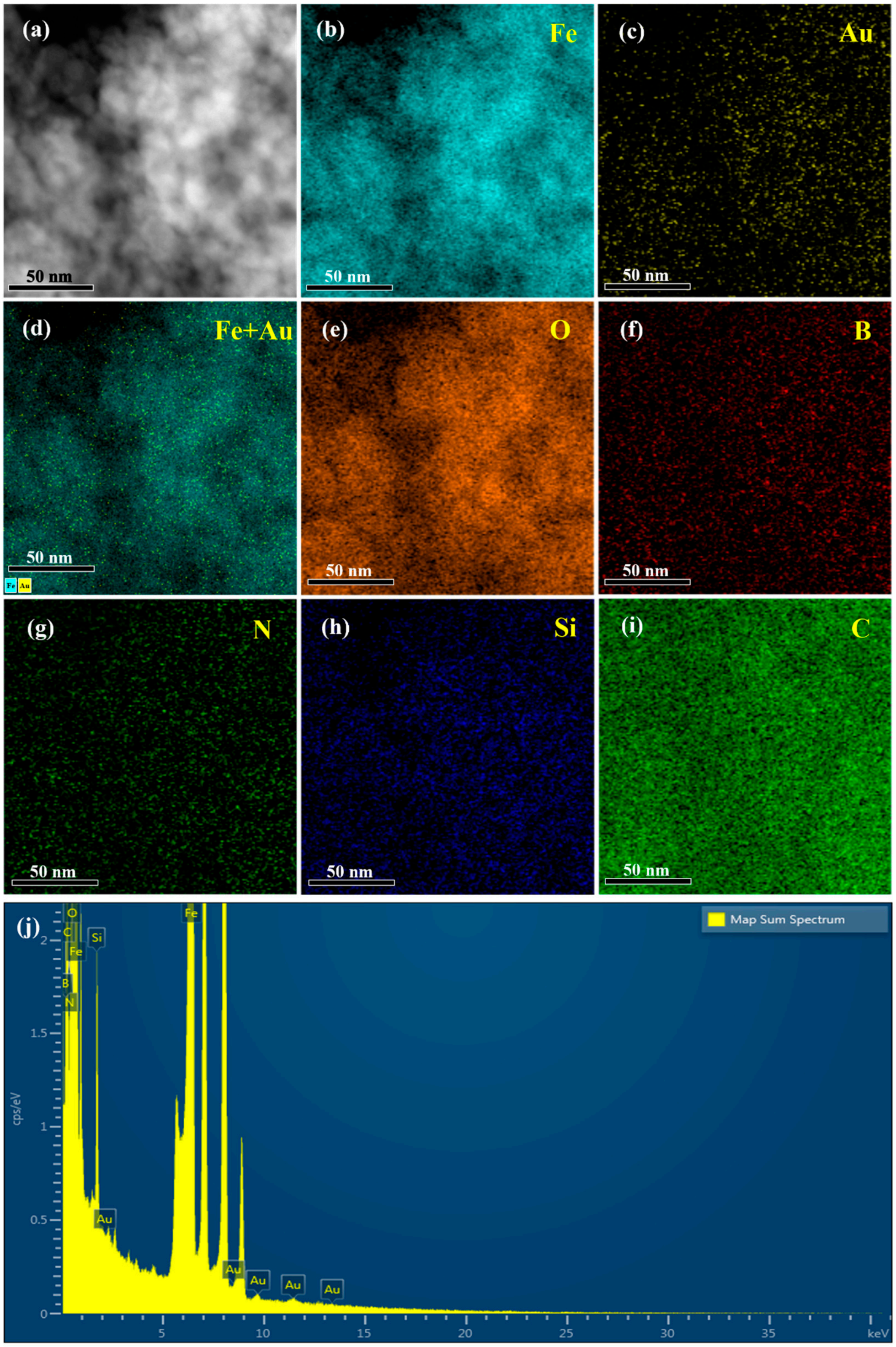

Figure S2 - STEM–EDS analysis of Fe_3O_4@Au–APTES–carborane particles. (a) TEM top-view image of the Fe_3O_4@Au–APTES–carborane particles. (b–i) STEM–EDS elemental maps of the region shown in (a) for Fe, Au, O, B, N, Si, and C. (j) Corresponding summed EDS spectrum of the analyzed elements. The STEM–EDS elemental mapping clearly demonstrates the uniform distribution of Au on the surface of the Fe_3O_4 particles. The high carbon content is associated with the use of a carbon-based mesh in the TEM grid.

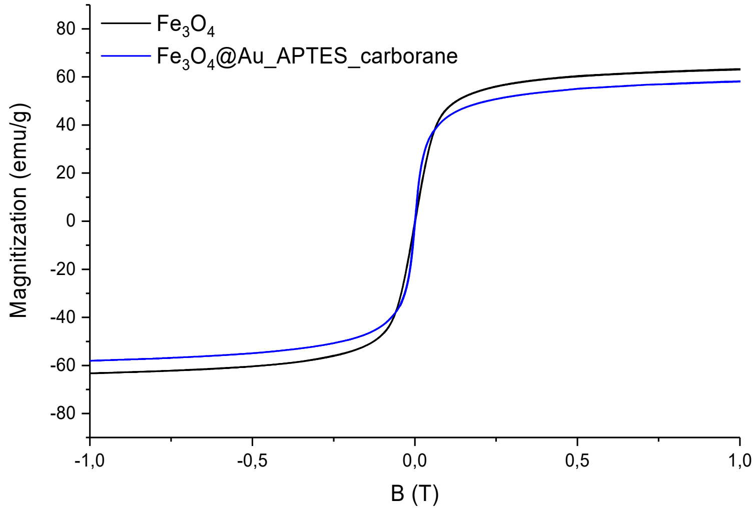

Figure S3 - Hysteresis loops of Fe_3O_4 and Fe_3O_4@Au–APTES–carborane nanoparticles.

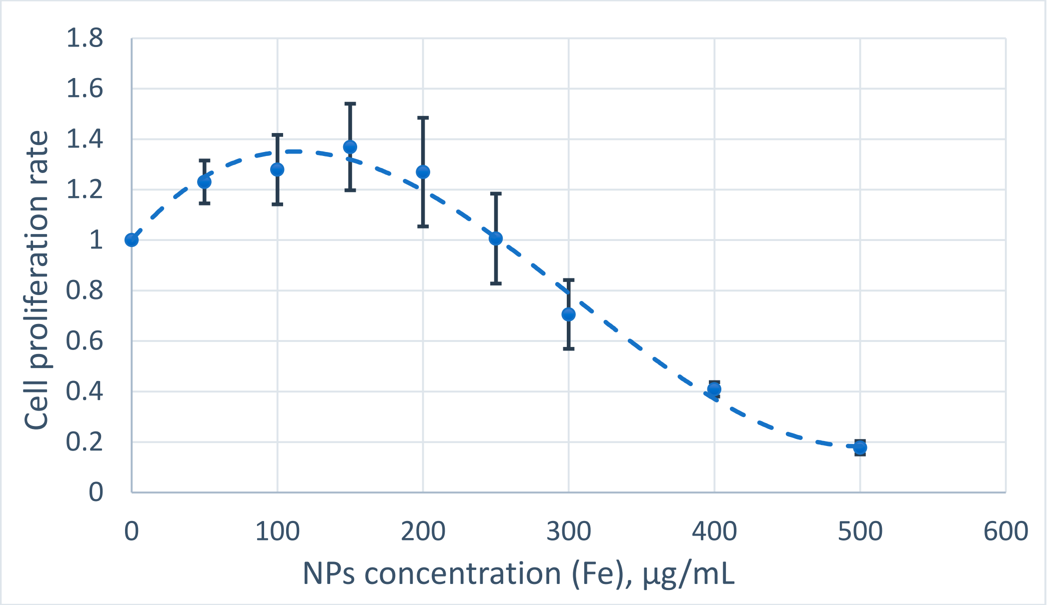

Figure S4 - Proliferation of primary mouse astrocyte (AWT) cells after 24-hour incubation with Fe_3O_4@Au–APTES–carborane nanoparticles. AWT cells were obtained from the RIKEN Cell Bank (Cell Line No. RCB5681; RIKEN BRC, Tsukuba, Ibaraki, Japan) and cultured under conditions similar to those used for U251MG cells, as described in the manuscript.
